# Impact of insurance coverage on utilization of pre-exposure prophylaxis for HIV prevention

**DOI:** 10.1371/journal.pone.0178737

**Published:** 2017-05-30

**Authors:** Rupa R. Patel, Leandro Mena, Amy Nunn, Timothy McBride, Laura C. Harrison, Catherine E. Oldenburg, Jingxia Liu, Kenneth H. Mayer, Philip A. Chan

**Affiliations:** 1 Division of Infectious Diseases, Washington University in St. Louis, St. Louis, Missouri, United States of America; 2 Division of Infectious Diseases, University of Mississippi Medical Center, Jackson, Mississippi, United States of America; 3 Department of Behavioral and Social Sciences, Brown University School of Public Health, Providence, Rhode Island, United States of America; 4 Brown School and Center for Health Economics and Policy, Washington University in St. Louis, St. Louis, Missouri, United States of America; 5 Francis I. Proctor Foundation, University of California San Francisco, San Francisco, California, United States of America; 6 Division of Public Health Sciences, Washington University in St. Louis, St. Louis, Missouri, United States of America; 7 Division of Infectious Diseases, Beth Israel Deaconess Medical Center, Boston, Massachusetts, United States of America; 8 Department of Medicine, Harvard Medical School, Boston, Massachusetts, United States of America; 9 The Fenway Institute, Fenway Health, Boston, Massachusetts, United States of America; 10 Division of Infectious Diseases, Brown University, Providence, Rhode Island, United States of America; University of Rhode Island, UNITED STATES

## Abstract

Pre-exposure prophylaxis (PrEP) can reduce U.S. HIV incidence. We assessed insurance coverage and its association with PrEP utilization. We reviewed patient data at three PrEP clinics (Jackson, Mississippi; St. Louis, Missouri; Providence, Rhode Island) from 2014–2015. The outcome, PrEP utilization, was defined as patient PrEP use at three months. Multivariable logistic regression was performed to determine the association between insurance coverage and PrEP utilization. Of 201 patients (Jackson: 34%; St. Louis: 28%; Providence: 28%), 91% were male, 51% were White, median age was 29 years, and 21% were uninsured; 82% of patients reported taking PrEP at three months. Insurance coverage was significantly associated with PrEP utilization. After adjusting for Medicaid-expansion and individual socio-demographics, insured patients were four times as likely to use PrEP services compared to the uninsured (OR: 4.49, 95% CI: 1.68–12.01; p = 0.003). Disparities in insurance coverage are important considerations in implementation programs and may impede PrEP utilization.

## Introduction

In 2014, over 40,000 individuals were diagnosed with human immunodeficiency virus (HIV) infection in the United States (US) with the majority among men who have sex with men (MSM) [1]. There are large disparities in HIV incidence, with African Americans facing a disproportionate burden of new infections [1]. Pre-exposure prophylaxis (PrEP) refers to the use of antiretrovirals to prevent HIV infection prior to exposure. It is estimated that over 1.2 million adults in the US are at risk for HIV and would benefit from PrEP [2]. Taken as a daily single oral tablet, PrEP has been proven to prevent HIV infection in MSM and other populations [3,4]. PrEP implementation has become a national priority to reduce new HIV infections [5,6].

One potential barrier to wider PrEP use in the US is insurance coverage. Insurance coverage usually leads to increased access to healthcare and use of healthcare services [7]. However, 17.9% of adults ages 25–34 years were uninsured in 2015, despite the passage of the Affordable Care Act [8]. Few studies have evaluated whether insurance coverage impacts PrEP care [5]. We previously evaluated retention-in-care outcomes among patients enrolled in PrEP programs in three US cities; insurance was not found to affect PrEP care and retention among a subanalysis of MSM at these sites [9]. Recent data from participants in the US PrEP Demonstration Project showed that individuals who were non-white, uninsured, and of lower socioeconomic status reported barriers to accessing PrEP after the Demonstration Project ended [10]. White participants, in addition to those with health insurance, higher education, older age, and reported willingness to pay more for PrEP, were more likely to have continued PrEP after study completion [10]. These differences in PrEP use by race and socioeconomic status could potentially exacerbate existing disparities in HIV incidence.

To further explore the impact of insurance coverage on PrEP utilization in the US, we evaluated whether insurance status was associated with PrEP utilization among patients prescribed PrEP at clinics in three states with varied Medicaid expansion [11].

## Methods

We reviewed patients in clinical PrEP programs in Providence, Rhode Island, Jackson, Mississippi and St. Louis, Missouri from January 2014 to December 2015. In Rhode Island, PrEP patients were seen at a sexually transmitted diseases and HIV prevention clinic that was academically affiliated; patients were referred to the clinic from an onsite HIV clinic and other providers [9]. In Mississippi, PrEP patients were seen at a lesbian/gay/bisexual/transgender outpatient clinic; patients were referred from other providers and the state sexually transmitted diseases clinic [9]. In Missouri, PrEP patients were seen at an academic infectious diseases specialty clinic; referrals to this clinic were from community-based organizations, local outpatient providers, and an onsite HIV clinic [9]. All three sites were the major outpatient PrEP providers in their respective states. This analysis was a sub-study of an ongoing prospective observational cohort [9]. Inclusion criteria were patients who were prescribed PrEP for at least three months. Demographic and insurance-related information was collected during clinical visits as part of routine patient care procedures and data was extracted from the electronic medical records. Demographic data included age, gender, race, ethnicity, education, income, and insurance type. Insurance type included having no insurance or having private or public insurance (i.e. government-funded) [9].

As a part of the Affordable Care Act, state governments could choose to expand Medicaid eligibility to those with annual incomes below 138% of the federal poverty level [12]. Nineteen U.S. states have not yet expanded Medicaid. At the time of this study, Rhode Island was the only Medicaid-expansion state among our three sites. We created an indicator variable for state Medicaid expansion and grouped study states according to this status. Insurance status was further categorized by whether patients who had either private or public insurance. Private insurance included individual or group commercial insurance. Public insurance was defined as Medicaid, Medicare, or another public insurance program.

This study was approved by the Institutional Review Boards at Washington University in St. Louis, University of Mississippi, and The Miriam Hospital for human subject’s research. All participants provided written informed consent.

### Statistical analysis

The primary outcome was PrEP utilization, defined as patient report of taking PrEP at their three-month follow-up evaluation. Evaluations were performed in person as part of a routine clinical care visit or over the phone, when a physical appointment was not possible; routine clinical care was administered as per national guidelines [9]. Independent variables included age, race, education, income, insurance status, state Medicaid expansion, and study site. We created a Medicaid state expansion variable to account for the fact that in states with Medicaid expansion, it may be easier to acquire insurance, which may affect care utilization [13,14]. The association between independent variables and the outcome was assessed using chi-square or Fisher’s exact tests. To investigate factors predicting the primary outcome, univariate and multivariate logistic regression models were used. Odds ratios (OR) and 95% confidence intervals (CI) were calculated. We assessed the impact of insurance on PrEP utilization using a multivariable logistic regression Model 1 and adjusted for age, race, education, income, and individual insurance coverage. In subsequent models, we also assessed how insurance was affected by the addition of state Medicaid expansion (Model 2) and study site (Model 3). We accounted for Medicaid expansion since this affects the ability to obtain insurance for people within specific states. To control for the study site within the analysis, we looked at study site in two ways: 1) with Mississippi as a referent (versus Missouri and Rhode Island) and 2) with each state separately with Rhode Island as a referent. All statistical tests were two-sided and the significance level was set at 0.05.

## Results

Overall, 201 PrEP patients were included into the analysis (33.8% from Mississippi, 27.9% from Missouri, and 38.3% from Rhode Island). Eighty-two percent of patients reported taking PrEP at three months.

Patient characteristics included median age of 29 years (Interquartile Range [IQR] 24–37), 91.0% were male, 51.2% were white, 34.3% were African American, 6.0% were Latino, 64.7% were college graduates, 79.1% had either public or private insurance, and median income was $25,000 (IQR $7,200-$50,000) (Table 1). Almost all patients (95%) were insured in Providence, 49% in Jackson and 95% in St. Louis (*P*<0.01).

**Table 1:** Study population characteristics (N = 201).

Characteristic	Missed 3-month Appointment N = 36	Attended 3-month Appointment N = 165	P value
Gender			0.34^†^
Male	31 (86.1)	152 (92.1)	
Female	5 (13.9)	12 (7.3)	
Transgender	0 (0.0)	1 (0.6)	
Age (Years)			**0.02**
18–24	15 (41.7)	36 (31.8)	
25–34	9 (25.0)	75 (45.5)	
>34	12 (33.3)	54 (32.7)	
Race			0.09^†^
White	15 (41.7)	88 (53.3)	
African American	18 (50.0)	51 (30.9)	
Latino	0 (0.0)	12 (7.3)	
Mixed/Other	3 (8.3)	14 (8.5)	
Education			0.91
< College	13 (36.1)	58 (35.2)	
≥ College	23 (63.9)	107 (64.8)	
Income			0.13
< $25000	22 (61.1)	78 (47.3)	
≥ $25000	14 (38.9)	87 (52.7)	
Insurance			**0.003**
None	15 (41.7)	27 (16.4)	
Public	4 (11.1)	24 (14.5)	
Private	17 (47.2)	114 (69.1)	
State Medicaid Expansion*			0.65
No	21 (58.3)	103 (62.4)	
Yes	15 (41.7)	62 (37.6)	
Site			0.10
Providence	15 (41.7)	62 (37.6)	
St. Louis	5 (13.9)	51 (30.9)	
Jackson	16 (44.4)	52 (31.5)	

^†^Fisher’s Exact Test

*Medicaid Expansion State: Rhode Island

Univariate and multivariate logistic regression analyses are shown in Table 2. In the initial multivariate logistic regression analysis model (Model 1), which included age, race, education, income and individual insurance coverage, having insurance was significantly and positively associated with PrEP utilization (OR = 3.48, 95% CI: 1.39–8.69). Insurance remained significant when adjusting for state Medicaid expansion (Model 2) as well as for study site (Model 3) (OR = 4.49, 95% CI: 1.68–12.01 and OR = 3.98, 95% CI: 1.36–11.63, respectively). None of the other independent variables were found to be significant predictors. When looking at the effect of the study site using Jackson, Mississippi as a referent on the outcome, there were no significant findings in univariate or multivariate analysis (i.e. adjusting for demographics, insurance, and study site) (unadjusted OR = 1.74, 95% CI: 0.83–3.63 and adjusted OR = 0.79, 95% CI: 0.29–2.18). The significance of insurance was not affected by this re-categorization of study site using Jackson, Mississippi as the referent (OR = 4.15, 95% CI: 1.49–11.59).

**Table 2:** Univariate and multivariate logistic regression analysis for HIV pre-exposure prophylaxis utilization among 201 patients in three US cities (Jackson, Mississippi; St. Louis, Missouri; Providence, Rhode Island) from January 2014 to December 2015.

Characteristic	Unadjusted OR (95% CI)	Model 1* aOR (95% CI)	Model 2** aOR (95% CI)	Model 3*** aOR (95% CI)
Age Group				
< 30 years	1.0	1.0	1.0	1.0
≥ 30 years	1.21 (0.58–2.49)	0.92 (0.41–2.10)	0.95 (0.41–2.18)	0.95 (0.41–2.19)
Race				
White	1.0	1.0	1.0	1.0
Not White	0.63 (0.30–1.30)	0.99 (0.40–2.47)	0.86 (0.34–2.19)	0.90 (0.35–2.35)
Education				
< College	1.0	1.0	1.0	1.0
≥ College	1.04 (0.49–2.21)	0.87 (0.38–1.98)	0.81 (0.36–1.85)	0.82 (0.36–1.88)
Income				
< $25000	1.0	1.0	1.0	1.0
≥ $25000	1.75 (0.84–3.66)	1.19 (0.49–2.90)	1.23 (0.50–3.00)	1.21 (0.50–2.97)
Insurance				
No	1.0	1.0	1.0	1.0
Yes (public or private)	**3.65 (1.67–7.97)**	**3.48 (1.39–8.69)**	**4.49 (1.68–12.01)**	**3.98 (1.36–11.63)**
State Medicaid Expansion^†^				
No	1.0	-	1.0	-
Yes	0.84 (0.41–1.76)	-	0.46 (0.19–1.10)	-
Site				
Providence	1.0	-	-	1.0
St. Louis	2.47 (0.84–7.25)	-	-	2.58 (0.86–7.70)
Jackson	0.79 (0.36–1.74)	-	-	1.82 (0.62–5.38)

Abbreviations: OR, odds ratio; aOR, adjusted OR

*Adjusted for age, race, education, income, and individual insurance coverage

**Adjusted for age, race, education, income, individual insurance coverage, and state Medicaid expansion

***Adjusted for age, race, education, income, individual insurance coverage, and study site

^†^Medicaid Expansion State: Rhode Island

## Discussion

We found that having insurance coverage may significantly impact PrEP utilization. Insured patients in our sample were four times as likely to use PrEP services compared to the uninsured. Our results should be taken into the context that nearly one out of five young adults are uninsured and that national HIV burden is highest in the southern US, where non-Medicaid expansion limits insurance coverage eligibility [1,8]. Furthermore, in the context of upcoming proposed changes to the healthcare system, our findings indicate that there could be potential setbacks in PrEP implementation if reforms make accessing insurance more difficult [14,15]. The proposed American Health Care Act would cause 14 million people to lose their insurance in 2018 and 24 million over the next decade [15,16]. Specific reforms include changing the federal match for Medicaid to a block grant policy where the government caps the amount of money allotted per Medicaid enrollee and would match only 50% of costs per enrollee versus the 90% under the Affordable Care Act [14,15]. This change in Medicaid policy would cause 17 million Americans to lose their insurance within 10 years. Other reforms that have the potential to change insurance access or quality of coverage to pay for PrEP-related care and costs for young adults, a group with high HIV incidence, include changing a mandate for employers to provide insurance as well as a $7 billion reduction in federal subsidies to reduce deducible and copayment costs for care for enrollees. Inadequate insurance coverage for at-risk individuals undermines the goal of reducing HIV incidence through PrEP and other preventative care.

Since Medicaid eligibility expansion increases the ability to obtain insurance despite variations in patient backgrounds (i.e. race, income, age, etc.), we evaluated this concept within our analysis. In this sample, adjusting for Medicaid expansion using multiple logistic regression did not diminish insurance coverage’s association with PrEP utilization. This study is limited by the inclusion of only one Medicaid expansion state; however our analyses highlighted private insurance’s effect on PrEP utilization given the effect of insurance remained significant after controlling separately for Medicaid expansion status in the model. Further larger studies should explore Medicaid expansion’s impact on PrEP use. Such findings could help influence retaining stronger preventive care policies within the context of today’s health care policy reforms.

Prior studies have demonstrated that insurance coverage influences PrEP care [5,10]. Among patients in The Demo Project in San Francisco, 11.8% discontinued PrEP due to leaving their health plan. Prior work has demonstrated that a significant facilitator to PrEP uptake is free access [17]. A modeling study conducted in Georgia, a Medicaid non-expansion state, among 562 MSM predicted that only 15% of MSM would achieve protection from HIV by using PrEP largely due to significant barriers to health care access, which was defined as receiving a prescription and paying provider visits and laboratory-related costs [18]. Although the manufacturer of PrEP, Gilead Sciences, has a patient drug assistance program, the current study demonstrates that ongoing structural gaps may impede PrEP use by those who might benefit most [19]. The findings are particularly notable, given that the patients in this study received care in specialized clinics, with staff available to help them navigate the paperwork needed to access insurance and/or medication.

Limitations of the study include a small sample size, a large portion of uninsured patients came from one site (MS), and that insurance coverage was examined at one time point (i.e. initial evaluation). Future studies should include other clinical settings, rural populations, more states with varying Medicaid expansion, a method to capture insurance mobility, and evaluate insurance coverage’s effects on other aspects of PrEP care. Examining these factors will provide important insights for PrEP program planners, policy makers, and others on how to effectively improve PrEP implementation, especially for individuals with financial barriers and who lack insurance.

Insurance coverage had a significant and positive impact on PrEP utilization. Disparities in insurance coverage may impede PrEP implementation in non-trial clinical settings and may exacerbate disparities in PrEP access. These findings from three diverse areas suggest state- and local-level PrEP implementation efforts should address barriers to insurance coverage as a critical component to successful programmatic efforts.

## Supporting information

S1 File Insurance and retention in PrEP care dataset. (XLSX)

## References

[1] Centers for Disease Control and Prevention. Diagnoses of HIV infection in the United States and dependent areas, 2014. HIV surveillance report 2015;26. 2014.

[2] Smith DK, Van Handel M, Wolitski RJ, Stryker JE, Hall HI, Prejean J, et al Vital Signs: Estimated Percentages and Numbers of Adults with Indications for Preexposure Prophylaxis to Prevent HIV Acquisition—United States, 2015. MMWR Morb Mortal Wkly Rep. 2015;64(46):1291–1295. doi: 10.15585/mmwr.mm6446a4 26606148 10.15585/mmwr.mm6446a4

[3] Grant RM, Lama JR, Anderson PL, McMahan V, Liu AY, Vargas L, et al Preexposure chemoprophylaxis for HIV prevention in men who have sex with men. NEJM. 2010;363(27):2587–2599. doi: 10.1056/NEJMoa1011205 21091279 10.1056/NEJMoa1011205 PMC3079639

[4] McCormack S, Dunn DT, Desai M, Dolling DI, Gafos M, Gilson R, et al Pre-exposure prophylaxis to prevent the acquisition of HIV-1 infection (PROUD): effectiveness results from the pilot phase of a pragmatic open-label randomised trial. Lancet. 2016;387(10013):53–60. doi: 10.1016/S0140-6736(15)00056-2 26364263 10.1016/S0140-6736(15)00056-2 PMC4700047

[5] Liu A, Cohen S, Follansbee S, Cohan D, Weber S, Sachdev D, et al Early experiences implementing pre-exposure prophylaxis (PrEP) for HIV prevention in San Francisco. PLoS Med. 2014;11(3):e1001613 doi: 10.1371/journal.pmed.1001613 24595035 10.1371/journal.pmed.1001613 PMC3942317

[6] The White House. National HIV/AIDS Strategy for the United States: Updated to 2020. July 2015. Accessed from: https://www.whitehouse.gov/administration/eop/onap/nhas Accessed on: September 27, 2016.

[7] Long SK, Coughlin T, King J. How well does Medicaid work in improving access to care? Health Serv Res. 2005;40(1):39–58. doi: 10.1111/j.1475-6773.2005.00341.x 15663701 10.1111/j.1475-6773.2005.00341.x PMC1361125

[8] Cohen RA MM, Zammitti EP. Health insurance coverage: Early release of estimates from the National Health Interview Survey, 2015. National Center for Health Statistics 5 2016.

[9] Chan PA, Mena L, Patel R, Oldenburg CE, Beauchamps L, Perez-Brumer AG, et al Retention in care outcomes for HIV pre-exposure prophylaxis implementation programmes among men who have sex with men in three US cities. J Int AIDS Soc. 2016;19(1):20903 doi: 10.7448/IAS.19.1.20903 27302837 10.7448/IAS.19.1.20903 PMC4908080

[10] Doblecki-Lewis S, Liu A, Feaster D, Cohen SE, Cardenas G, Bacon O, et al Healthcare Access and PrEP Continuation in San Francisco and Miami Following the U.S. PrEP Demo Project. J Acquir Immune Defic Syndr. 2016. 10.1097/QAI.0000000000001236 PMC5340616 27861236

[11] Health and Human Services. Compilation of the Patient Protection and Affordable Care Act. May 1, 2010. Available at: https://www.hhs.gov/sites/default/files/ppacacon.pdf

[12] U.S. Centers for Medicare & Medicaid Services. Accessed from: www.healthcare.gov. Accessed on: September 27, 2016.

[13] Miller S, Wherry LR. Health and Access to Care during the First 2 Years of the ACA Medicaid Expansions. N Engl J Med. 2017;376(10):947–956. doi: 10.1056/NEJMsa1612890 28273021 10.1056/NEJMsa1612890

[14] Goodman-Bacon AJ, Nikpay SS. Per Capita Caps in Medicaid—Lessons from the Past. N Engl J Med. 2017;376(11):1005–1007. doi: 10.1056/NEJMp1615696 28146644 10.1056/NEJMp1615696

[15] Congressional Budget Office. American Health Care Act. March 13, 2017. https://www.cbo.gov/publication/52486.

[16] Library of Congress. H.R.1628- American Health Care Act of 2017. https://www.congress.gov/bill/115th-congress/house-bill/1628/text

[17] Golub SA, Gamarel KE, Rendina HJ, Surace A, Lelutiu-Weinberger CL. From efficacy to effectiveness: facilitators and barriers to PrEP acceptability and motivations for adherence among MSM and transgender women in New York City. AIDS Patient Care STDs. 2013;27(4):248–254. doi: 10.1089/apc.2012.0419 23565928 10.1089/apc.2012.0419 PMC3624632

[18] Kelley CF, Kahle E, Siegler A, Sanchez T, Del Rio C, Sullivan PS, et al Applying a PrEP Continuum of Care for Men Who Have Sex With Men in Atlanta, Georgia. Clin Infect Dis. 2015;61(10):1590–7. doi: 10.1093/cid/civ664 26270691 10.1093/cid/civ664 PMC4614414

[19] Gilead [internet]. Truvada for PrEP Medication Assistance Program [cited 2017 May 13]. Available from: http://www.gilead.com/responsibility/us-patient-access/truvada%20for%20prep%20medication%20assistance%20program

